# USP15 inhibits multiple myeloma cell apoptosis through activating a feedback loop with the transcription factor NF-κBp65

**DOI:** 10.1038/s12276-018-0180-4

**Published:** 2018-11-20

**Authors:** Lili Zhou, Hua Jiang, Juan Du, Lu Li, Rong Li, Jing Lu, Weijun Fu, Jian Hou

**Affiliations:** 1 Shanghai Jiahui International Hospital Cancer Center, Shanghai, 200233 China; 2 Department of Hematology, Shanghai Changzheng Hospital, Second Military Medical University, Shanghai, 200433 China; 3 Department of Hematology, Renji Hospital affiliated to Shanghai Jiaotong University School of Medicine, Shanghai, 200127 China

## Abstract

USP15 has been shown to stabilize transcription factors, to be amplified in many cancers and to mediate cancer cell survival. However, the underlying mechanism by which USP15 regulates multiple myeloma (MM) cell proliferation and apoptosis has not been established. Here, our results showed that USP15 mRNA expression was upregulated in MM patients. USP15 silencing induced MM cell proliferation inhibition, apoptosis, and the expression of nuclear and cytoplasmic NF-κBp65, while USP15 overexpression exhibited an inverse effect. Moreover, in vivo experiments indicated that USP15 silencing inhibited MM tumor growth and NF-κBp65 expression. PDTC treatment significantly inhibited USP15 overexpression-induced cell proliferation, apoptosis inhibition, and NF-κBp65 expression. USP15 overexpression promoted NF-κBp65 expression through inhibition of its ubiquitination, whereas NF-κBp65 promoted USP15 expression as a positive regulator. Taken together, the USP15-NF-κBp65 loop is involved in MM tumorigenesis and may be a potential therapeutic target for MM.

## Experimental & molecular medicine

Researchers may have found a new target for treatment of multiple myeloma (MM), a common type of blood cancer. MM develops in plasma cells, white blood cells that fight infection, and is very difficult to treat. Ubiquitins, small regulatory proteins which tag proteins for destruction, have been implicated in development of many cancers. Jian Hou at Ren Ji Hospital and Weijun Fu at Changzheng Hospital, both in Shanghai, China, investigated how one ubiquitin, USP15, is involved in MM. Patients with MM showed higher levels of USP15, and MM cells supplied with additional USP15 multiplied more rapidly. Treating MM mice with USP15 inhibitors slowed tumor growth. Further investigation showed that USP15 interacts with another molecule known to be involved in development of many cancers. These results may help to develop alternative treatments for multiple myeloma.

## Introduction

Multiple myeloma (MM) is a malignant blood system disease derived from B cells. It is characterized by clonal expansion of plasma cells in the bone marrow, which secretes a large number of monoclonal immunoglobulins, together with a series of dissolved bone lesions, clinical symptoms, and organ dysfunction, such as bone disease, pathological fractures, renal failure, and anemia^1,2^. MM constitutes approximately 1% of all malignant tumors and is the second most common blood system tumors, surpassed only by lymphoma^3^. The MM mortality is as high as 70–90%. Since the pathogenesis of MM is complex, the number and structural abnormalities of chromosomes, activation of oncogenes, inactivation of tumor suppressors, IL-6-dependent cytokine network disorders, and changes in bone marrow microenvironment are all related to the occurrence of myeloma^4,5^. With the application of proteasome inhibitors and immunomodulators, the therapeutic efforts in MM patients have improved^6^. The 5 and 10-year survival rates of patients with MM were increased from 32.8 and 15% to 40.3 and 20.8%, respectively^7^. However, because of many problems such as multidrug resistance and associated side effects, MM is still an incurable hematologic tumor. Therefore, it is important to further study the molecular mechanism and find more potential therapeutic targets for the treatment of MM.

Ubiquitination is a post-translational protein modification process that connects single or multiple ubiquitin molecules to a target protein and affects its stability and function. Deregulation of the deubiquitination process is frequently associated with tumorigenesis^8,9^. Ubiquitin-specific proteases (USPs) are deubiquitinating enzymes that reverse the ubiquitination through removing ubiquitin from the targeted proteins by directly interacting with substrates or indirectly binding to an adaptor protein such as E3 ubiquitin ligase. USP15 functions with the E3 ubiquitin ligase TRIM25 to positively regulate type I interferon responses and to promote pathogenesis during neuroinflammation^10^. USP15 also regulates certain mutant versions of p53 and binds to and stabilizes p53 through deubiquitination in osteosarcoma and ovarian cancer cells^11,12^. Reduced accumulation of IκB-α after its TNF-α-induced degradation was observed in HeLa cells with suppression of USP15 expression, suggesting nuclear translocation of NF-κB in TNF-α-stimulated cells^13^. Additionally, USP15 silencing also abolished the inhibitory effect of morphine on NF-κB signaling^14^. However, the correlation between USP15 and NF-κB and the effect of USP15 on apoptosis in MM are still unclear.

The highly abnormal and persistently activated NF-κB is associated with the proliferation, cell cycle process, apoptosis, metabolism, and drug resistance of MM^15,16^. The ubiquitination process is involved in the activation of the NF-κB pathway through degradation of IκB-α and activation of IκB kinase. Regulation of the ubiquitination process therefore directly affects the activation of NF-κB^17^. In this study, we have evaluated the biological functions of USP15 in apoptosis and proliferation of MM cells and the underlying molecular mechanisms involved. Upregulation of USP15 was in MM patients was found to induce cell proliferation and inhibit cell apoptosis of MM through activating NF-κB signaling. USP15 promoted NF-κBp65 expression through inhibiting ubiquitination. USP15 inhibited MM cell apoptosis through activating a feedback loop with NF-κBp65.

## Materials and methods

### Clinical samples

Ninety-five cases of bone marrow samples from 80 patients with MM and 15 patients with proliferative bone marrow (PBM) were collected in Changzheng Hospital from March 2011 to May 2017. Written informed consent was obtained from all participants in this study. The study protocol was approved by the ethics committee of Changzheng Hospital.

### Cell culture

RPMI 8226, U266, H929, KMS12, and KMS18 human MM cell lines obtained from the Cell Bank of the Chinese Academy of Science (Shanghai, China) and non-cancerous bone marrow-derived plasma cells (control) were cultured in RPMI-1640 medium (Hyclone, USA) containing 10% fetal bovine serum (GIBCO) and 1% antibiotic (mixtures of penicillin and streptomycin, Solarbio) in a 37 °C, 5% CO_2_ incubator (Thermo, USA). The old medium was replaced with fresh medium depending on the growth of the cells during the period of culture.

### Cell transfection

Two siRNAs targeting human USP15 (point 1, 1077-1095, 5′-GAGGTGAAATAGCTAAATC-3′; point 2, 1754-1772, 5′-GATACAGAGCACGTGATTA-3′) were produced and transfected into the RPMI 8226 and U266 cells using Lipofectamine 2000 (Invitrogen, USA) following the manufacturer’s protocol. The coding sequence of USP15 was synthesized using the primers containing the restriction enzyme cut sites of *Bam*HI and *Eco*RI (USP15 Forward: 5′-GCGAATTCATGGCGGAAGGCGGAGCGGCGGAT-3′ and Reverse: 5′-CGGGATCCTTAGTTAGTGTGCATACAGTTTTC-3′) and integrated into the pLVX-Puro plasmid to increase USP15 expression. Recombinant plasmids along with the psPAX2 and pMD2G packaging plasmids were co-transfected into 293 T cells using Lipofectamine 2000 (Invitrogen, USA). Forty-eight h after transfection, recombined lentiviral vectors were collected and used to infect RPMI 8226 and U266 cells. Cells with scrambled siRNA (siNC) and blank pLVX-Puro (Vector) transfection were used as negative control.

### CCK-8 assay

The Cell Counting Kit (CCK)-8 (Beyotime, Shanghai, China) was used to examine MM cell proliferation. Briefly, cells (5 × 10^3^ cell/well) cultured in complete medium containing the NF-κB inhibitor PDTC (50 μM) or no inhibitor were processed following the standard procedure in 96-well plates and were maintained in 5% CO_2_ incubator at 37 °C overnight. After 0, 24, 48 and 72 h transfections of RPMI 8226 and U266 cells with siUSP15#1, siUSP15#2 or pLVX-Puro-USP15, the CCK-8 solution (10 μl per well) was added to the cells which were then maintained in a CO_2_ incubator for 1 h at 37 °C, after which the absorbance readings were obtained at 450 nm.

### Cell apoptosis assay

In accordance with the manufacturer’s instructions, the apoptosis of MM cells was analyzed using the Annexin V-FITC/Propidium Iodide (PI) cell apoptosis kit (BD Biosciences, USA). RPMI 8226 and U266 cells cultured in complete medium containing PDTC (50 μM) or no inhibitor were transfected with siUSP15#1, siUSP15#2 or pLVX-Puro-USP15 for 48 h, washed three times with PBS, digested by trypsin, centrifuged (1000x*g*, room temperature) for 10 min, adjusted to 5 × 10^5^ cell/ml and suspended in the Annexin V-FITC and PI-binding buffer. The apoptotic cells were measured with flow cytometry (BD Biosciences) after 10 min of incubation in the dark.

### Luciferase reporter experiments

RPMI 8226 and U266 cells (5 × 10^5^ cell/well) cultured in complete medium containing PDTC (50 μM) or the NF-κB-agonist LPS (1 μg/ml) were seeded in a 6-well plate, cultured in an incubator with 5% CO_2_ at 37 °C for 24 h, and then transfected with 1.5 μg of the pGL3-basic plasmid containing the USP15 promoter at 37 °C for 6 h using Lipofectamine 2000 (Invitrogen) following the manufacturer’s protocol. Forty-eight h after transfection, 100 μl of the luciferase assay reagent and 10 μl of the Stop&Glo reagent were added into the RPMI 8226 and U266 cells. Luciferase activity (Firefly and Renilla) was measured with the Dual-Luciferase Reporter assay system (Promega) according to the manufacturer’s protocol.

### Real-time PCR

We applied Real-time PCR to detect the mRNA levels of USP15, Caspase-3, PARP1, Bcl-2, Bcl-xL and Survivin. First, extraction of total RNA from MM tissues and cell lines was achieved by TRIzol (Invitrogen). Subsequently, the TaqMan reverse transcription kit (Applied Biosystems, USA) was used to reverse the isolated RNA into cDNA. Real-time PCR was performed using the SYBR Green qRT-PCR kit (Promega, USA) on an ABI7500 system following the manufacturer’s instructions to analyze the mRNA levels normalizing to GAPDH. The following are the primer sequences: USP15, 5′-TGCCTACTTCCAACTCTC-3′ and 5′-GCTCTTCCTTTCCTTCTC-3′; Caspase-3, 5′-TGGTTCATCCAGTCGCTTTG-3′ and 5′-AAATTCTGTTGCCACCTTTCG-3′; PARP1, 5′-TCACGGACACGCTTTCACC-3′ and 5′-CCCCGCAGATTCTACATTCG-3′; Bcl-2, 5′-GCAGTGTGGTCTCCGAATGTC-3′ and 5′-CATTGCCTCTCCTCACGTTCC-3′; Bcl-xL, 5′-CAGGTATGGAAGGGTTTG-3′ and 5′-TAGGGATGGAAGGAAAGG-3′; Survivin, 5′-CCACCGCATCTCTACATTC-3′ and 5′-CTTTCTCCGCAGTTTCCTC-3′; GAPDH, 5′-AATCCCATCACCATCTTC-3′ and 5′-AGGCTGTTGTCATACTTC-3′. The relative expression of mRNAs was calculated through the 2^-ΔΔCT^ method.

### Western blotting

Total protein was extracted using a total protein extraction buffer (Beyotime, China). A 10% sodium dodecyl sulfate polyacrylamide gel was prepared to isolate the proteins. After transferred to a nitrocellulose membrane, the protein bands were blocked with 5% non-fat milk. The blots were then incubated with primary antibodies and secondary antibodies. The antibodies and reagents used were as follows: USP15 (Abcam, ab71713, 1:1000); active Caspase-3 (Abcam, ab2302, 1:200); cleaved PARP1 (Abcam, ab32064, 1:1000); Survivin (Abcam, ab76424, 1:3000); Bcl-2 (Abcam, ab32124, 1:1000); Bcl-xL (Abcam, ab32370, 1:1000); NF-κBp65 (Cell Signaling Technology, #8242, 1:1000); IκB-α (Cell Signaling Technology, #9242, 1:1000); p-IκB-α (Cell Signaling Technology, #2859, 1:1000); H3 (Cell Signaling Technology, #4499S, 1:2000); GAPDH (Cell Signaling Technology, #5174, 1:2000); HRP-labeled Goat Anti-Rabbit IgG (Beyotime, A0208, 1:1000); HRP-labeled Donkey Anti-Goat IgG (Beyotime, A0181, 1:1000); and HRP-labeled Goat Anti-Mouse IgG (Beyotime, A0216, 1:1000). Signals were detected using an enhanced chemiluminescence Western Blotting substrate (Pierce; Thermo Fisher Scientific, Inc., USA).

### Co-immunoprecipitation (Co-IP) and ubiquitination in vitro

Co-IP was performed as previously described^18^. Briefly, cold PBS was used to wash the cells three times, and the cells were scraped into lysis buffer containing complete protease inhibitors and centrifuged at 14,000×*g* for 20 min at 4 °C. The supernatants were incubated with anti-NF-κBp65 (1:1000), anti-IκB-α (1:1000) or normal IgG (1:1000) antibody, and the immunocomplexes were then associated with protein A-sepharose. Anti-NF-κBp65 (1:1000), anti-IκB-α (1:1000) and anti-ubiquitin (1:2000) antibodies were used for western blot analysis.

### Animal experiments

For the tumor growth assays in vivo, RPMI 8226 cells with pLKO.1-USP15-shRNA or negative control (shNC) transfection were resuspended in PBS at a concentration of 1 × 10^7^ cells/ml. The cell suspension (100 μl) was injected into the right armpit of BALB/c male nude mice (4–5 week-old; 6 per group). Forty-eight days after injection, the mice were sacrificed, and tumor tissues were excised and weighed. Animal experiments were performed according to the legal requirements and were approved by the Changzheng Hospital institutional ethical committee.

### Statistical analysis

Data are presented as the mean ± SD, and each test was repeated at least three times. Statistical analysis was conducted using one-way ANOVA with GraphPad Prism software, version 5 (GraphPad Software, USA). *P* < 0.05 was regarded as statistically significant.

## Results

### USP15 mRNA expression in MM tissues and cell lines

**Fig. 1:**
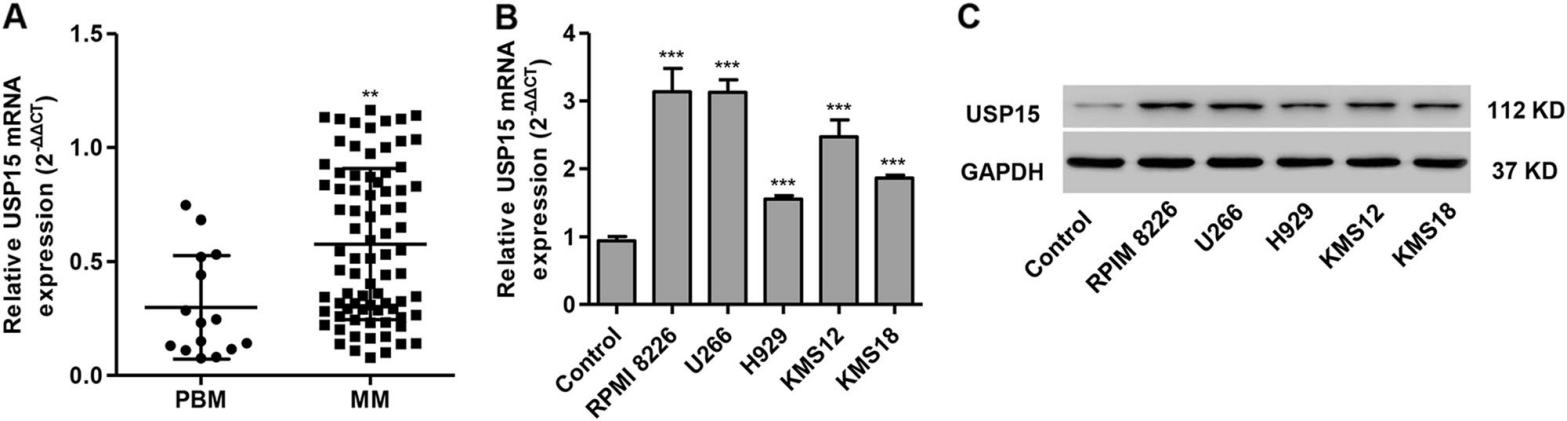
USP15 expression in MM tissues and cell lines. **a** The mRNA expression of USP15 in bone marrow samples from 80 patients with multiple myeloma (MM) and 15 patients with proliferative bone marrow (PBM) was measured by Real-time PCR. The expression of USP15 in five different MM cell lines and in control cells, non-cancerous bone marrow-derived plasma cells, was measured by Real-time PCR (**b**) and western blotting (**c**). **P < 0.01 compared with PBM or control

The mRNA expression of USP15 in the bone marrow of patients with MM (*n* = 80) was upregulated compared to patients with PBM; (*n* = 15) (Fig. 1a). Moreover, the mRNA expression of USP15 in five different MM cell lines and in control cells, which were non-cancerous bone marrow-derived plasma cells, was also measured. We found that USP15 expression was higher in MM cell lines compared with control cells, with the highest expression detected in RPMI 8226 and U266 cells compared with other MM cell lines (Fig. 1b, c). These results suggest that USP15 may participant in the development and progression of MM.

### USP15 overexpression promotes MM cell proliferation

**Fig. 2:**
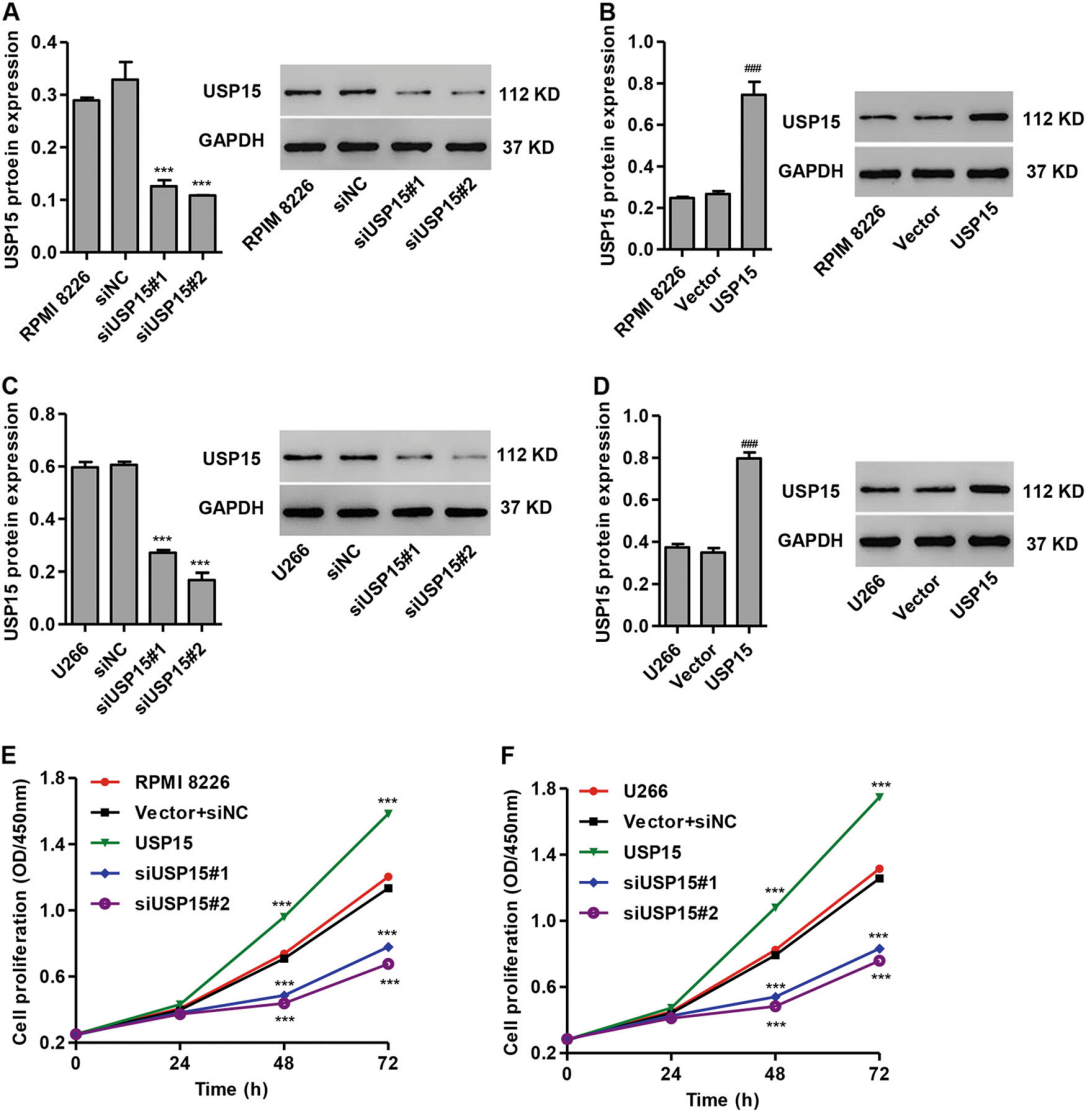
Effects of USP15 RNA-interference and USP15 overexpression on MM cell proliferation. After RPMI 8226 and U266 cells were transfected with siNC plus empty pLVX-Puro (vector), siUSP15#1, siUSP15#2, or pLVX-Puro-USP15, the expression of USP15 was measured by western blot (**a**–**d**) and cell proliferation was measured by the CCK-8 assay (**e**, **f**). ***P < 0.001 compared with siNC, vector or vector + siNC

To evaluate the role of USP15 in MM tumorigenesis in vitro, RPMI 8226 and U266 cells were transfected with siUSP15#1, siUSP15#2, or pLVX-Puro-USP15. As shown in Fig. 2a, siUSP15#1 and siUSP15#2 transfection in RPMI 8226 cells significantly reduced USP15 protein expression by 61.7% and 66.9% compared to siNC, respectively. siUSP15#1 and siUSP15#2 transfection in U266 cells significantly reduced USP15 protein expression by 55.2% and 72.2% compared to siNC, respectively (Fig. 2c). pLVX-Puro-USP15 transfection in RPMI 8226 and U266 cells significantly increased USP15 protein expression by 1.79-fold and 1.28-fold compared to the blank vector, respectively (Fig. 2b, d).

Furthermore, the CCK-8 assay demonstrated that siUSP15#1 and siUSP15#2 transfection in RPMI 8226 cells significantly inhibited cell proliferation by 30.3% and 37.2%, respectively, at 48 h after transfection compared to the vector plus siNC group (Fig. 2e). siUSP15#1 and siUSP15#2 transfection in U266 cells significantly inhibited cell proliferation by 31.0% and 38.2%, respectively, at 48 h after transfection compared to the vector plus siNC group (Fig. 2f). pLVX-Puro-USP15 transfection in RPMI 8226 and U266 cells significantly increased cell proliferation by 35.6% and 36.5%, respectively, at 48 h after transfection compared to the vector plus siNC group (Fig. 2e, f). These data indicate that USP15 has a pro-proliferative effect in MM cells.

### USP15 overexpression inhibits MM cell apoptosis

**Fig. 3:**
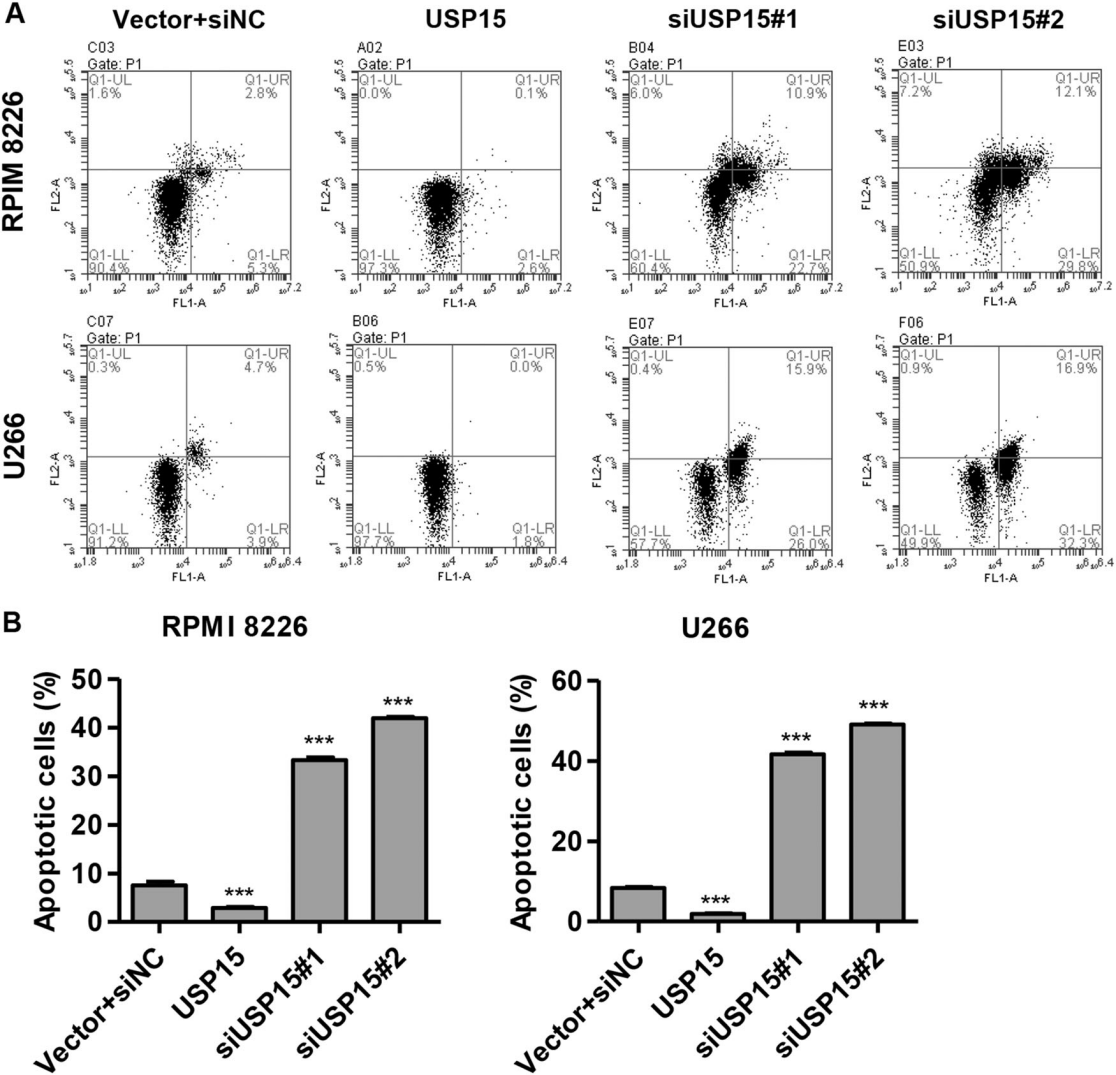
USP15 overexpression inhibits MM cell apoptosis. **a**, **b** After RPMI 8226 and U266 cells were transfected with siNC plus empty pLVX-Puro (vector), siUSP15#1, siUSP15#2, or pLVX-Puro-USP15 for 48 h, apoptosis in the RPMI 8226 and U266 cells was measured by flow cytometry with Annexin V-FITC/PI staining. ***P < 0.001 compared with vector + siNC

Flow cytometry analysis was performed to examine the effect of USP15 on MM cell apoptosis. We found that siUSP15#1 and siUSP15#2 transfection in RPMI 8226 and U266 cells significantly promoted cell apoptosis compared to the vector plus siNC group (Fig. 3a, b). pLVX-Puro-USP15 transfection in RPMI 8226 and U266 cells significantly inhibited cell apoptosis compared to the vector plus siNC group. These results suggest that USP15 has an anti-apoptotic effect in MM cells.

### USP15 overexpression promotes the expression of Bcl-2, Bcl-xL, Survivin, and NF-κBp65

**Fig. 4:**
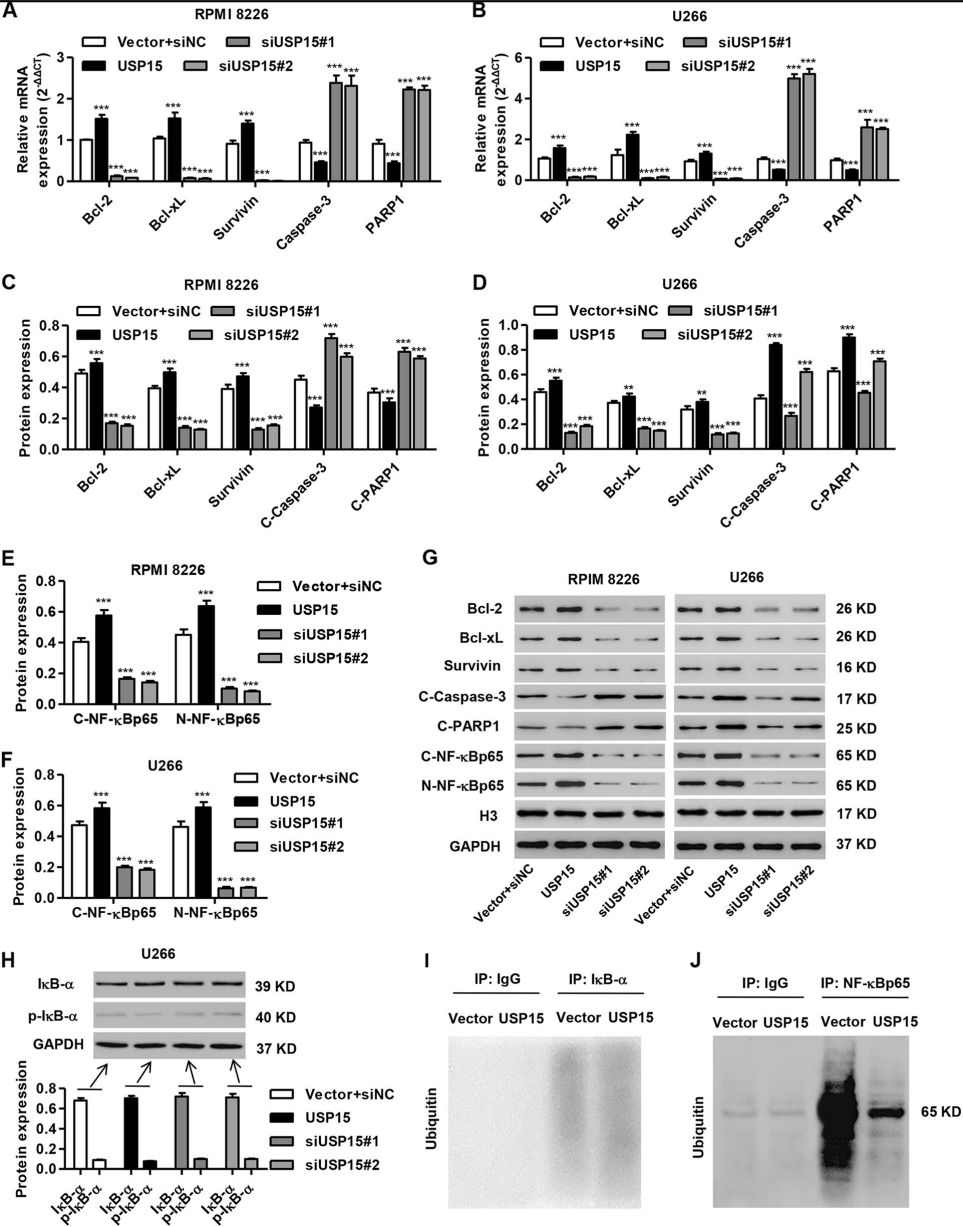
USP15 overexpression promotes the expression of Bcl-2, Bcl-xL, Survivin, and NF-κBp65. After RPMI 8226 and U266 cells were transfected with siNC plus empty pLVX-Puro (vector), siUSP15#1, siUSP15#2, or pLVX-Puro-USP15, the expression of Bcl-2, Bcl-xL, Survivin, cleaved Caspase-3 (C-Caspase-3), cleaved PARP1 (C-PARP1), nuclear NF-κBp65, and cytoplasmic NF-κBp65 was measured by Real-time PCR (**a**, **b**) and/or western blot (**c**-**g**). (**h**) IκB-α and p-IκB-α expression in U266 cells was measured by western blot. IκB-α (**i**) or NF-κBp65 (**j**) was immunoprecipitated and immunoblotted in cells with empty pLVX-Puro (vector) or pLVX-Puro-USP15 transfection. ***P < 0.001 compared with vector + siNC

To investigate the molecular mechanism of USP15 in the regulation of proliferation and apoptosis in MM cells, the expression of Caspase-3, PARP1, Bcl-2, Bcl-xL, Survivin, nuclear NF-κBp65, and cytoplasmic NF-κBp65 was measured by real-time PCR and/or western blot. As shown in Fig. 4a, b, siUSP15#1 and siUSP15#2 transfection in RPMI 8226 and U266 cells significantly reduced the mRNA expression of Bcl-2, Bcl-xL and Survivin but induced Caspase-3 and PARP1 mRNA expression compared to the vector plus siNC group. Further, transfection with siUSP15#1 and siUSP15#2 also mediated the expression of these proteins and inhibited nuclear NF-κBp65 and cytoplasmic NF-κBp65 expression (Fig. 4c–g). However, pLVX-Puro-USP15 transfection had an inverse effect on expression of these proteins. Given the effects of USP15 on NF-κBp65 expression, the role of USP15 in the expression and ubiquitination of IκB-α was also measured. As shown in Fig. 4h and I, USP15 overexpression and silencing had no effect on the expression of IκB-α and p-IκB-α in U266 cells, and its overexpression did not affect IκB-α ubiquitination, which suggests that USP15 may regulate NF-κBp65 expression in an IκB-α-independent manner. Additionally, USP15 overexpression significantly inhibited the ubiquitination of NF-κBp65 (Fig. 4j). These results suggest that USP15 promotes NF-κBp65 expression through deubiquitination.

### USP15 knockdown inhibits MM tumor growth and protein expression in vivo

**Fig. 5:**
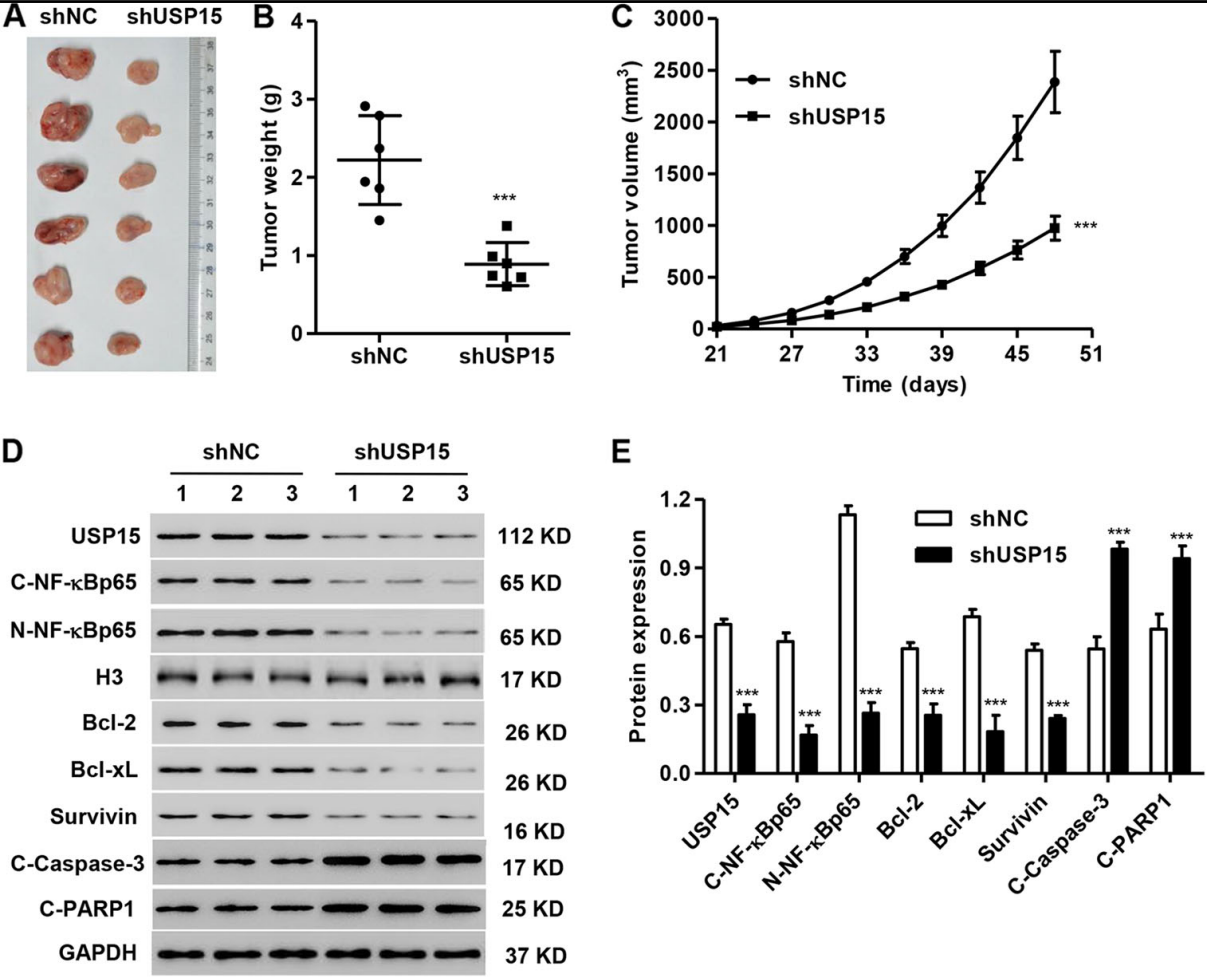
**USP15 knockdown inhibits tumor growth and related protein expression in MM in vivo.** After RPMI 8226 cells were transfected with pLKO.1-USP15-shRNA or the negative control (shNC) and injected into the nude mouse xenograft model, the mice were sacrificed 48 days later, the tumor weight (**a**, **b**) and volume (**c**) were evaluated, and the expression of USP15, Bcl-2, Bcl-xL, Survivin, cleaved Caspase-3 (C-Caspase-3), cleaved PARP1 (C-PARP1), nuclear NF-κBp65, and cytoplasmic NF-κBp65 in xenograft tumors was measured by western blot (**d**, **e**). ***P < 0.001 compared with shNC

To evaluate the function of USP15 in MM in vivo, RPMI 8226 cells transfected with pLKO.1-USP15-shRNA or shNC were injected into nude mice. We found that mice with pLKO.1-USP15-shRNA injection exhibited decreased tumor weight and tumor volume (Fig. 5a–c). The expression of USP15, Bcl-2, Bcl-xL, Survivin, nuclear NF-κBp65, and cytoplasmic NF-κBp65 was significantly decreased, but the expression of cleaved Caspase-3 and PARP1 was increased in xenograft tumors with pLKO.1-USP15-shRNA injection compared to the shNC injection (Figs. 5d, e).

### PDTC treatment inhibits USP15 overexpression-induced MM cell proliferation and apoptosis inhibition

**Fig. 6:**
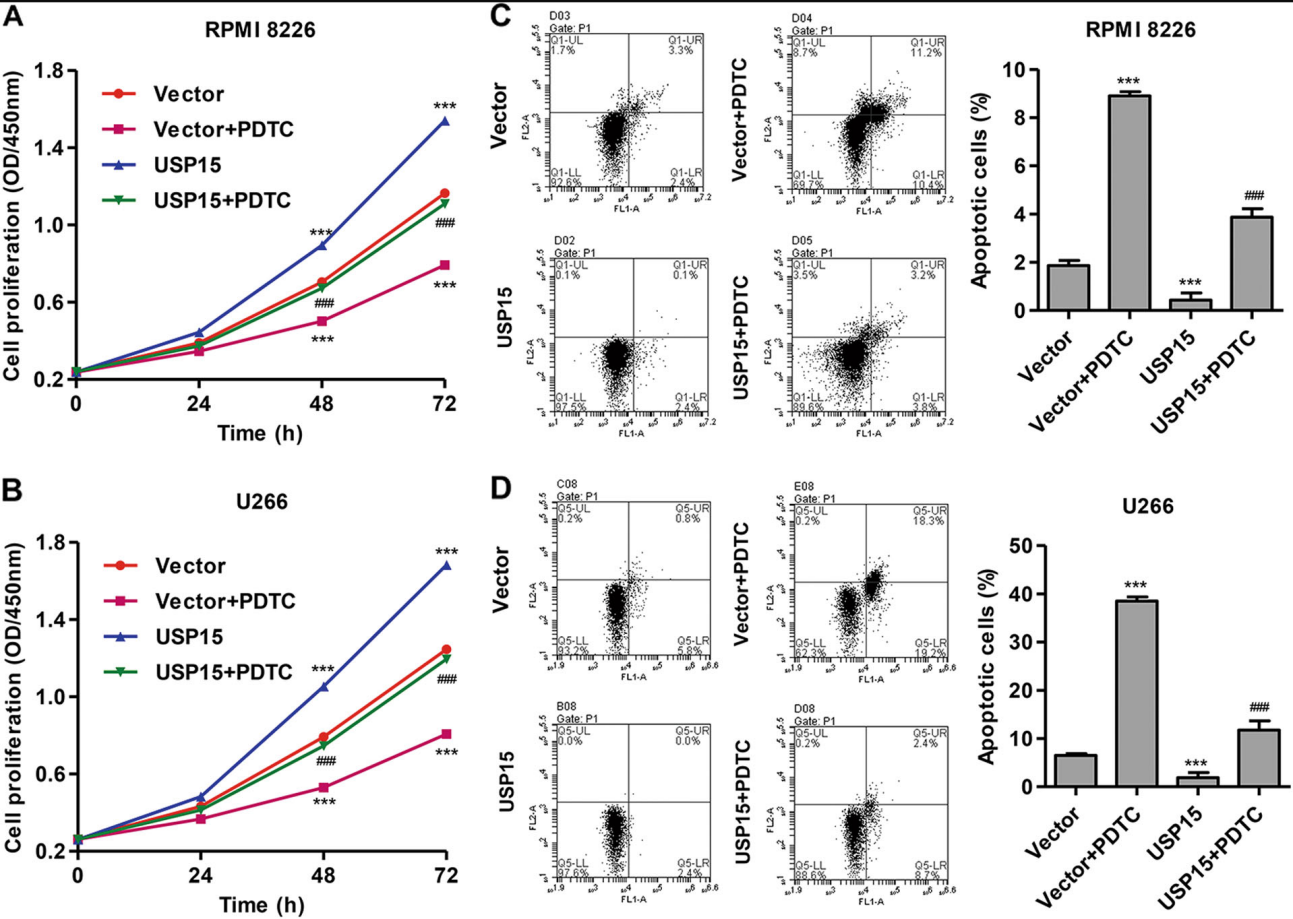
**PDTC treatment inhibits USP15 overexpression-induced cell proliferation and apoptosis inhibition in MM.** After RPMI 8226 and U266 cells cultured in complete medium containing either PDTC (50 μM) or no inhibitors were transfected with pLVX-Puro-USP15, proliferation was measured with the CCK-8 assay (**a**, **b**) and apoptosis was measured by flow cytometry with Annexin V-FITC/PI staining (**c**, **d**). ***P < 0.001 compared with vector. ^###^*P* < 0.001 compared with USP15

**Fig. 7:**
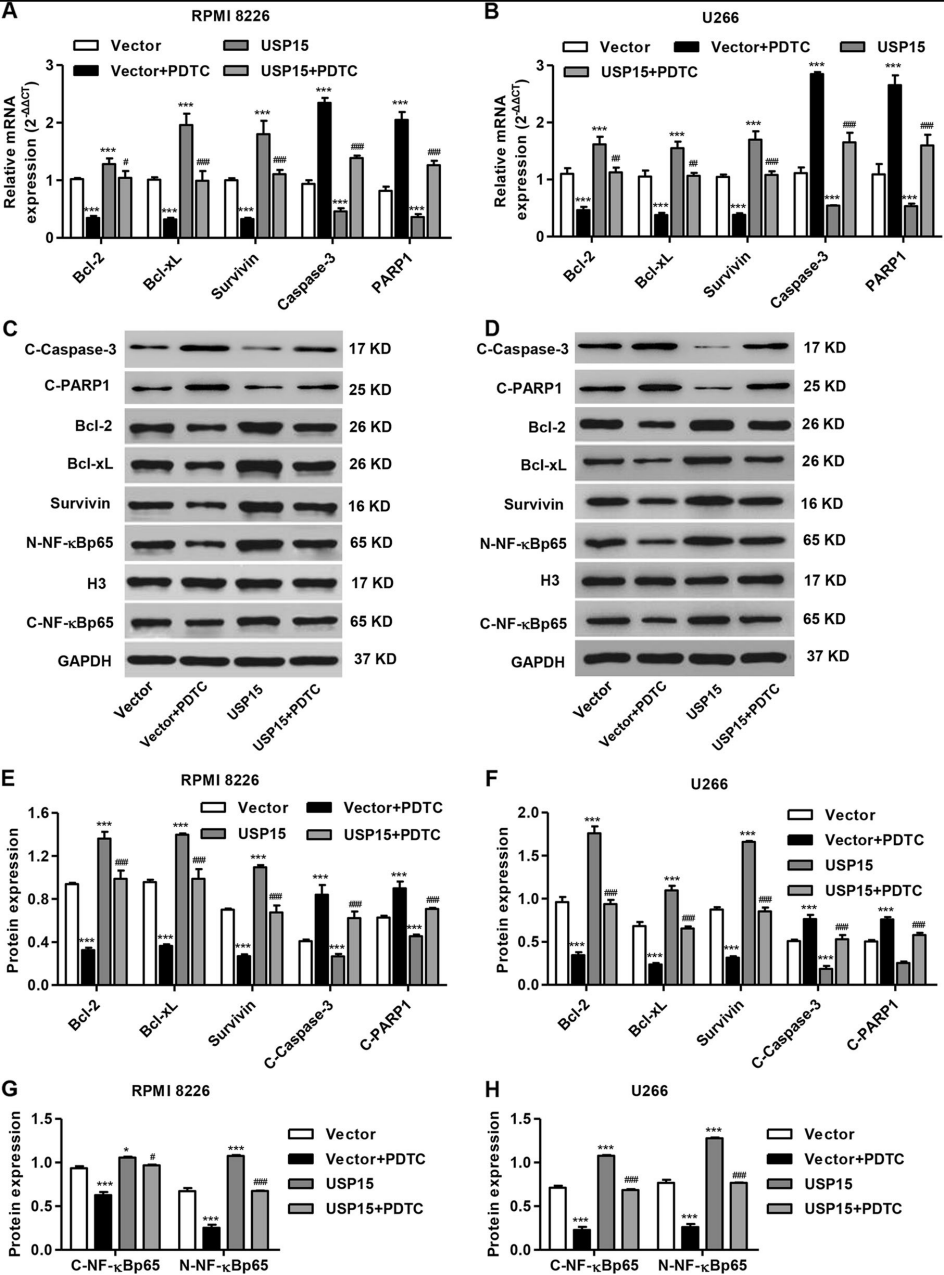
PDTC treatment inhibits USP15 overexpression-induced expression of Bcl-2, Bcl-xL, Survivin and NF-κBp65 in MM cells. After RPMI 8226 and U266 cells cultured in complete medium containing either PDTC (50 μM) or no inhibitor were transfected with pLVX-Puro-USP15, the expression of Bcl-2, Bcl-xL, Survivin, cleaved Caspase-3 (C-Caspase-3), cleaved PARP1 (C-PARP1), nuclear NF-κBp65, and cytoplasmic NF-κBp65 was measured by Real-time PCR (**a**, **b**) and/or western blot (**c**-**h**). *P < 0.05, ***P < 0.001 compared with vector. ^#^*P* < 0.05; ^##^*P* < 0.01; ^###^*P* < 0.001 compared with USP15

To investigate the role of NF-κBp65 signaling in USP15-mediated MM cell proliferation and apoptosis, the NF-κBp65 signaling inhibitor PDTC was added to MM cell cultures. As shown in Fig. 6a, b, PDTC treatment significantly inhibited the USP15 overexpression-induced proliferation of RPMI 8226 and U266 cells by 24.9 and 29.2% at 48 h after transfection, respectively. PDTC treatment also significantly inhibited the USP15 overexpression-induced decrease in apoptosis of RPMI 8226 and U266 cells by 7.9-fold and 5.3-fold, respectively (Fig. 6c, d). Moreover, the expression of Bcl-2, Bcl-xL, Survivin, nuclear NF-κBp65, and cytoplasmic NF-κBp65 was significantly decreased, and the expression of cleaved Caspase-3 and PARP1 was increased in MM cells after PDTC treatment (Fig. 7). These results suggest that USP15 regulates MM cell proliferation and apoptosis through NF-κBp65 signaling.

### Evidence of an NF-κBp65-USP15 regulatory loop in MM cells

**Fig. 8:**
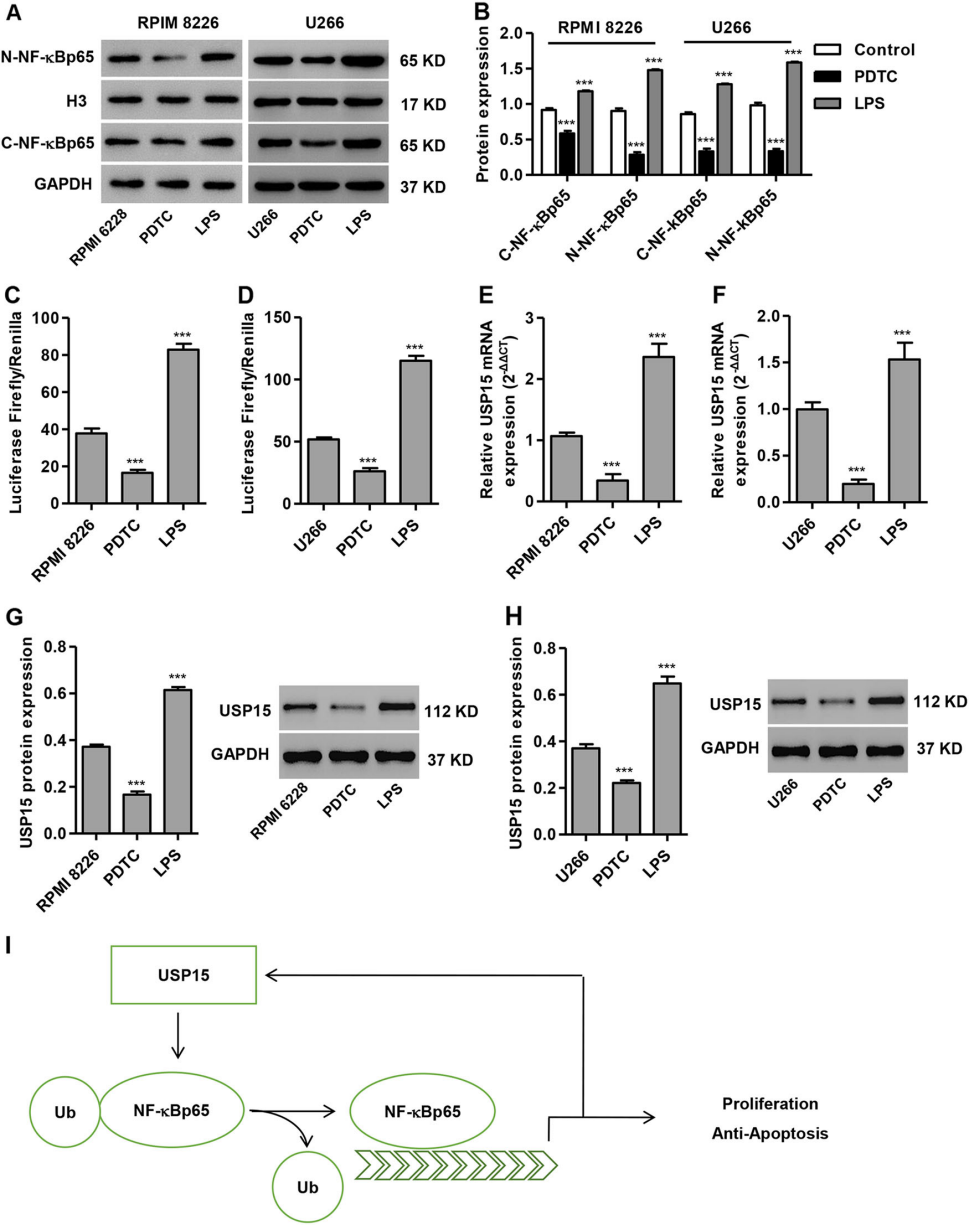
Regulatory feedback between USP15 and NF-κBp65. **a**, **b** After RPMI 8226 and U266 cells were treated with PDTC (50 μM) or LPS (1 μg/ml), the expression of nuclear NF-κBp65 and cytoplasmic NF-κBp65 was measured by western blot. **c**, **d** Dual-luciferase assays in RPMI 8226 and U266 cells transfected with firefly luciferase constructs containing the 3′ UTR of USP15 with or without PDTC (50 μM) and LPS (1 μg/ml) treatment. After RPMI 8226 and U266 cells were treated with PDTC (50μM) or LPS (1 μg/ml), the expression of USP15 was measured by Real-time PCR (**e**, **f**) and western blot (**g**, **h**). **i** Schematic representation USP15-NF-κBp65 regulation of cell proliferation and apoptosis. ***P < 0.001 compared with untreated control

Given the role of NF-κBp65 in transcriptional regulation, we evaluated the possible effect of NF-κBp65 on the regulation of USP15 mRNA by transfecting MM cells with a luciferase reporter vector containing the 3′UTR region of USP15 with or without PDTC (50 μM) or LPS (1 μg/ml) treatment. As shown in Fig. 8a, b, PDTC treatment significantly inhibited the expression of nuclear NF-κBp65 and cytoplasmic NF-κBp65, but LPS treatment promoted the expression of NF-κBp65 in RPMI 8226 and U266 cells. Whereas PDTC treatment significantly decreased the luciferase Firefly/Renilla ratio, LPS treatment significantly increased that ratio when compared to untreated cells, suggesting that NF-κBp65 enhances the promoter activity of USP15 (Fig. 8c, d). In addition, PDTC treatment in RPMI 8226 and U266 cells also significantly decreased USP15 mRNA and protein expression, but LPS treatment significantly increased their expression compared to untreated cells (Fig. 8e–h). These data indicate that the NF-κBp65-USP15 regulatory loop exists in MM cells.

## Discussion

Multiple myeloma (MM) is a malignant hematological tumor with monoclonal plasma cell hyperplasia, and it accounts for 10–15% of all blood system tumors. At present, combined chemotherapy is the main treatment for MM. Despite the continuous improvements in chemotherapy, this treatment does not cure the disease because many patients are unable to tolerate or resistant to conventional chemotherapy, and therefore experience a relapse or even death after a few months or several years^19,20^. In the present study, we found that USP15 mRNA expression was upregulated in the bone marrow of MM patients compared to patients with -PBM. Silencing USP15 inhibited MM cell growth both in vitro and in vivo. Overexpression of USP15 promoted proliferation and inhibited apoptosis, and these effects were inhibited by PDTC treatment. NF-κBp65 positively regulated USP15 expression, whereas USP15 overexpression promoted NF-κBp65 expression through deubiquitination of NF-κBp65 in MM cells. The USP15-NF-κBp65 regulatory loop was involved in MM cell apoptosis (Fig. 8i).

Increasing evidence has demonstrated that alterations in the deubiquitinating enzymes, including the ubiquitin-specific peptidase (USP) families, are implicated in the pathogenesis of a wide variety of tumors. A previous study showed that USP4 is upregulated in melanoma tissues and that it accentuates the migration and invasion of melanoma cells by promoting the epithelial-mesenchymal transition but has no effect on cell proliferation^21^. USP21 is upregulated in renal cell carcinoma tissues and cell lines, and depletion of USP21 inhibits cell proliferation and invasion through binding to the IL-8 promoter region and mediating transcriptional initiation^22^. USP14 is upregulated in oral squamous cell carcinoma tissues and cell lines; reducing USP14 levels inhibits cell viability and tumor growth in vivo and induces cell apoptosis in vivo but not in vitro^23^. These findings suggest that USPs may act as oncogenes associated with cancer tumorigenesis. In the present study, our results showed that USP15 is upregulated in MM patients and has pro-proliferative and anti-apoptotic roles in MM cells and xenograft tumors in nude mice, which is similar to the role in promoting oncogenesis observed in other cancers others^24,25^.

To investigate the molecular mechanism by which USP15 regulates MM cell proliferation and apoptosis, NF-κBp65 expression was also measured. NF-κBp65 expression was positively regulated by USP15 through inhibition of its ubiquitination in MM cells. The PDTC treatment-induced inhibition of NF-κBp65 in MM cells inhibited the USP15-induced reduction in apoptosis. Conversely, NF-κBp65 also enhanced the promoter activity of USP15 and its protein expression. Studies focused on the other USPs demonstrated that USP14 overexpression regulates IκB polyubiquitination to stimulate its degradation^26^, and ectopic expression of USP4 inhibits the TRAF2- and TRAF6-stimulated NF-κB reporter gene and negatively regulates the TNF-α-induced IκB-α degradation and NF-κB activation^27^. In this study, USP15 overexpression and silencing had no effect on the expression of IκB-α and p-IκB-α in U266 cells, and its overexpression did not affect IκB-α ubiquitination, which suggest that USP15 may regulate NF-κBp65 expression in an IκB-α-independent manner. However, USP15 regulates IκB-α ubiquitination and nuclear translocation of NF-κB in TNF-α-stimulated HeLa cells^13^. Overall, these data suggest an important role for TNF-α stimulation in the regulation of USP15-mediated expression and ubiquitination of IκB-α and NF-κB activity. Our data also demonstrated that PDTC treatment significantly inhibited the USP15-mediated expression of NF-κB-regulated gene products in MM cells. Inhibition of NF-κBp65 expression in MM cells significantly reduced proliferation and apoptosis and decreased Bcl-2 and Bcl-xL expression^28,29^. NF-κB inactivation decreases Bcl-2, Bcl-xL and Survivin expression and subsequently activates Caspase-3, which results in MM cell apoptosis^30,31^. These results indicate that USP15 regulates MM tumorigenesis through activating a feedback loop with NF-κBp65.

In conclusion, our results indicate that NF-κBp65 is involved in the regulation of USP15 in MM proliferation and apoptosis and that USP15 inhibits MM apoptosis through activating a feedback loop with NF-κBp65. Together, this suggests that USP15 may be a potential therapeutic target for MM.
